# Cardiovascular safety and early myocardial deformation signal after upadacitinib initiation in immune-mediated inflammatory disorders

**DOI:** 10.3389/fimmu.2026.1867314

**Published:** 2026-07-02

**Authors:** Salvatore Corrao, Salvatore Scibetta, Fabio Falcone, Ignazio Cangemi, Giacomo Corrao, Luigi Calvo

**Affiliations:** 1 Department of Clinical Medicine, Internal Medicine Unit with Rheumatology, Dermatology, Diabetology and Tertiary Diabetic Foot Health-Care, National Relevance and High Specialization Hospital Trust ARNAS Civico, Di Cristina, Benfratelli, Palermo, Italy; 2 Department of Health Promotion Sciences, Maternal and Infant Care, Internal Medicine and Medical Specialties (PROMISE), Palermo, Italy; 3 Institute for Biomedical Research and Innovation (IRIB), National Research Council (CNR), Palermo, Italy; 4 Department of Internal Medicine, Azienda Ospedaliera Universitaria “Policlinico G. Martino”, University of Messina, Messina, Italy

**Keywords:** cardiovascular safety, echocardiography, global longitudinal strain, immune-mediated inflammatory disorders, jak1, Janus kinase inhibitor, myocardial deformation, pharmacovigilance

## Abstract

**Objectives:**

Janus kinase (JAK) inhibitors are approved for several immune-mediated inflammatory disorders (IMIDs), but regulatory agencies, including the European Medicines Agency and the US Food and Drug Administration, have issued class-wide safety warnings regarding major adverse cardiovascular events, venous thromboembolism, and malignancy. Early myocardial effects after selective JAK1 inhibition have not been prospectively evaluated. We assessed cardiovascular safety and myocardial deformation after upadacitinib initiation in a real-world IMID cohort.

**Methods:**

In this prospective, single-centre, exploratory observational study, 36 consecutive adults with atopic dermatitis, prurigo nodularis, rheumatoid arthritis, or psoriatic arthritis initiating upadacitinib were enrolled. Transthoracic echocardiography with speckle-tracking global longitudinal strain (GLS) was performed at baseline, 1, 3, and 6 months. Echocardiographic operators were blinded to the study hypothesis. Longitudinal changes were analysed using rank-based repeated-measures models.

**Results:**

Thirty-six patients were included (median age 51 years; 55.6% female). Baseline left ventricular ejection fraction (LVEF) was preserved (median 56.0%, IQR 55.0–61.0), whereas GLS suggested subclinical myocardial dysfunction (−18.4%, IQR −19.8 to −16.2). No significant longitudinal changes were observed in LVEF (p=0.789), left ventricular mass index, relative wall thickness, or epicardial fat thickness, indicating no early structural or systolic harm. GLS showed a significant overall time effect (p=0.003), with a favourable trajectory at 1 month (p=0.003), sustained at 3 months (p=0.007) and 6 months (p=0.007). E/e′ increased modestly at 6 months (p=0.008) without structural remodelling.

**Conclusions:**

Upadacitinib initiation was not associated with early myocardial harm and showed a reassuring cardiovascular profile. A favourable GLS trajectory without LVEF change is hypothesis-generating and requires confirmation in adequately powered controlled studies.

## Highlights

- Upadacitinib initiation was not associated with early structural or systolic myocardial impairment.
- A favourable GLS trajectory emerged rapidly after treatment initiation and remained stable through 6 months.
- These findings support cardiovascular reassurance but require confirmation in larger controlled studies.

## Introduction

The cardiovascular safety of Janus kinase (JAK) inhibitors has emerged as a pivotal concern in clinical pharmacology and rheumatology. Following the results of the ORAL Surveillance trial, which demonstrated an increased risk of major adverse cardiovascular events (MACE) and malignancy with tofacitinib relative to tumour necrosis factor (TNF) inhibitors in patients with rheumatoid arthritis at elevated cardiovascular risk, the EMA and FDA issued class-wide safety restrictions for all approved JAK inhibitors (1, 2). These regulatory alerts recommend particular caution in patients aged ≥65 years, long-term smokers, and those with pre-existing cardiovascular or thromboembolic risk factors, and have substantially altered prescribing practice across all IMID indications. Patients with inflammatory rheumatic and immune-mediated conditions carry a well-documented excess cardiovascular burden that cannot be fully explained by traditional risk factors alone (3, 4). The cardiovascular burden is particularly relevant in psoriatic disease, where systemic inflammation and cardiometabolic comorbidity frequently coexist; recent data have confirmed a high prevalence of traditional cardiovascular risk factors among patients with psoriasis (5). Elevated cardiovascular morbidity and mortality have been demonstrated across the spectrum of IMIDs, including rheumatoid arthritis, psoriatic arthritis, systemic lupus erythematosus, and atopic dermatitis, among others (6). This residual risk is attributed primarily to chronic systemic inflammation, with ongoing cytokine activation driving endothelial dysfunction, vascular stiffness, metabolic dysregulation, and accelerated atherogenesis (7–10). The JAK/signal transducer and activator of transcription (JAK/STAT) pathway mediates the intracellular action of numerous pro-inflammatory cytokines central to IMID pathogenesis, including interleukins-6, -12, and -23 as well as interferons (8, 11). By targeting upstream intracellular signalling rather than individual extracellular mediators, JAK inhibitors may exert broad immunomodulatory effects; however, the cardiovascular consequences of this pharmacological approach require careful prospective characterisation. Four JAK inhibitors are currently approved for IMIDs: tofacitinib, baricitinib, upadacitinib, and filgotinib, each with a distinct isoform selectivity profile (11). Upadacitinib is a selective JAK1 inhibitor with demonstrated clinical efficacy across multiple IMID indications, including rheumatoid arthritis, psoriatic arthritis, ankylosing spondylitis, atopic dermatitis, and inflammatory bowel disease (12–21). In randomised trials, upadacitinib demonstrated superiority over placebo and non-inferiority or superiority to adalimumab across clinical, functional, and radiographic endpoints (16, 17). Nonetheless, the cardiovascular pharmacodynamic profile of upadacitinib in real-world practice — and in particular its early effects on subclinical myocardial function — remains poorly defined.

A critical and hitherto unaddressed dimension of JAK inhibitor cardiovascular safety concerns subclinical myocardial function. Global longitudinal strain (GLS), derived from two-dimensional speckle-tracking echocardiography, is a sensitive marker of early left ventricular systolic dysfunction, capable of detecting myocardial impairment before alterations in left ventricular ejection fraction (LVEF) become apparent (22–24). GLS retains independent prognostic value across multiple clinical settings, including heart failure with preserved ejection fraction (25, 26) and pharmacological cardiotoxicity monitoring (27). Current ESC guidelines on cardio-oncology endorse serial GLS assessment as a pharmacovigilance tool in patients receiving potentially cardiotoxic treatments (27). A comparable framework for early myocardial monitoring in patients initiating JAK inhibitor therapy has not previously been prospectively evaluated.

To date, no prospective study has systematically characterised early myocardial deformation changes following initiation of JAK inhibitor therapy. The present study was designed to assess the cardiovascular safety profile and the early pharmacodynamic myocardial deformation signal associated with upadacitinib initiation, using serial transthoracic echocardiography including speckle-tracking GLS, in a real-world cohort of IMID patients.

## Methods

### Study design and population

This was a prospective, single-centre, real-world observational cohort study conducted at the tertiary Immuno-Dermatology and Rheumatology Clinic of the Internal Medicine Unit, National Relevance and High Specialization Hospital Trust ARNAS Civico, Di Cristina, Benfratelli, Palermo, Italy. Consecutive patients initiating upadacitinib (Rinvoq^®^) were screened for inclusion. A total of 36 adults with established diagnoses of atopic dermatitis (UK Working Group criteria), prurigo nodularis, rheumatoid arthritis (ACR/EULAR 2010 criteria), or psoriatic arthritis (CASPAR criteria) were prospectively enrolled.

Inclusion criteria were age 18–64 years and provision of written informed consent. Exclusion criteria comprised heavy smoking (>20 pack-years), established cardiovascular disease, high or very high cardiovascular risk as estimated by SCORE2 or SCORE2-OP algorithms (28, 29), history of malignancy or pre-malignant lesions, active or chronic infections, and alcohol or drug abuse. The study was conducted in accordance with the Declaration of Helsinki and received approval from the local Ethics Committee of ARNAS Civico, Di Cristina, Benfratelli, Palermo, Italy (n. 46, prot. amm.vo n. 132 CIVICO 2025).

### Disease activity assessment

Disease-specific activity indices were recorded at baseline according to the underlying IMID subtype and routine clinical practice. These included the Eczema Area and Severity Index (EASI) for atopic dermatitis, the Disease Activity Score in 28 joints using C-reactive protein (DAS28-CRP) for rheumatoid arthritis, the Disease Activity index for Psoriatic Arthritis (DAPSA) for psoriatic arthritis, and the Bath Ankylosing Spondylitis Disease Activity Index (BASDAI) for axial/spondyloarthritic involvement. Because these instruments are disease-specific and not directly interchangeable across IMIDs, they were reported descriptively and were not pooled into a single longitudinal covariate.

### Echocardiographic acquisition

Transthoracic echocardiography was performed in accordance with the recommendations of the American Society of Echocardiography (ASE) and the European Association of Cardiovascular Imaging (EACVI) (30) using GE Vivid E95 scanners equipped with M5S-D phased-array transducers. All examinations were conducted with patients in the left lateral decubitus position; at least three cardiac cycles per acquisition were digitally recorded in DICOM format for offline analysis. Images were acquired at frame rates between 50 and 90 frames per second for speckle-tracking analysis. Strain measurements were analysed using EchoPAC SWO version 204 (GE HealthCare).

Left ventricular (LV) and left atrial (LA) volumes were measured using the modified biplane Simpson method from apical two- and four-chamber views, indexed to body surface area. Diastolic function assessment incorporated early (E) and late (A) mitral inflow velocities, deceleration time, tissue Doppler imaging-derived early diastolic velocity (E′) at the septal and lateral mitral annulus, and the mean E/e′ ratio. Epicardial fat thickness was measured in the parasternal long-axis view at end-systole on the free wall of the right ventricle and reported in millimetres.

Procedural blinding. Echocardiographic examinations were performed as an integral component of routine clinical patient monitoring, independently of the research protocol. At the time of image acquisition and offline analysis, echocardiographic operators were not informed of the specific study hypothesis or the expected direction of results. This procedural approach reduces the potential for observer bias during both acquisition and strain measurement, thereby strengthening the internal validity of the echocardiographic findings. Patient identifiers were removed before offline echocardiographic analysis and statistical processing; all analyses were conducted on anonymized data.

### Speckle-tracking strain analysis

Left ventricular GLS was evaluated using two-dimensional speckle-tracking echocardiography from apical long-axis, four-chamber, and two-chamber views, employing an 18-segment model. Manual adjustment of the region of interest was performed in all recordings; only studies with at least 16 of 18 segments adequately tracked were included in the analysis. End-systole was defined by aortic valve closure in the apical long-axis view, with consistent systolic timing applied across other apical views. GLS was reported as peak systolic whole-chamber strain, calculated by averaging all accepted segments.

Left atrial strain was analysed using the dedicated LA AFI package from LA-focused apical four-chamber views. The region of interest was manually adjusted to encompass the entire LA wall, excluding pulmonary veins and the LA appendage. The R-wave was used as the reference point in accordance with consensus recommendations (31). Reservoir strain values were used for all longitudinal analyses. All analyses were performed offline by experienced operators blinded to clinical data.

To assess reproducibility, GLS measurements were re-analysed in a random subset of patients. Intra- and inter-observer variability were quantified using intraclass correlation coefficients, demonstrating a high degree of reproducibility.

### Study endpoints

The primary endpoint was the longitudinal change in LV GLS following initiation of upadacitinib. Secondary echocardiographic endpoints comprised: LVEF, LV mass indexed to height^2.7^, relative wall thickness, mean E/e′ ratio, epicardial fat thickness, and LA reservoir strain. Echocardiographic evaluations were scheduled at baseline (T0) and at one (T1), three (T3), and six (T6) months, as clinically available.

### Statistical analysis

Continuous variables are expressed as median (interquartile range [IQR]); categorical variables are reported as number (percentage). Longitudinal analysis of echocardiographic variables was conducted using rank-based repeated-measures models to accommodate non-normal distributions and within-subject correlation. For each variable, observations were rank transformed across timepoints, with time treated as a categorical factor (T0, T1, T3, T6). Where numerical stability permitted, a rank-based mixed-effects model with a participant-specific random intercept was fitted. Where mixed-model estimation was unreliable (boundary solutions or singular covariance structures), inference was conducted using a rank-transformed linear model with participant fixed effects and cluster-robust standard errors.

The principal hypothesis test for each variable was the omnibus test of the overall time effect. Pre-specified pairwise comparisons versus baseline (T1 vs T0, T3 vs T0, T6 vs T0) were conducted within the same rank-based framework, with multiple testing controlled using the Holm procedure. A two-sided p-value <0.05 was considered statistically significant. Effect sizes were additionally reported as median paired changes. All analyses were performed using Python 3.11 (CPython), utilising the statsmodels, pandas, NumPy, and SciPy libraries.

No formal sample-size calculation was performed because of the absence of prior prospective data on serial GLS trajectories after initiation of JAK inhibitor therapy. The present study should therefore be considered exploratory and hypothesis-generating.

## Results

### Baseline characteristics

Thirty-six patients with chronic inflammatory diseases initiating upadacitinib were enrolled. Baseline demographic, clinical, laboratory, and echocardiographic features are summarised in **Table 1**. The median age was 51 years (IQR 33–60), and 55.6% of participants were female. The most prevalent index disease was atopic dermatitis or prurigo nodularis (55.6%), followed by rheumatoid arthritis (27.8%) and psoriatic arthritis or spondyloarthritis (16.7%). Median disease duration was 8.5 years (IQR 4.0–22.5). The cardiometabolic profile reflected a moderate burden of traditional risk factors: arterial hypertension was present in 33.3%, dyslipidaemia in 27.8%, and type 2 diabetes mellitus in 11.1% of participants. Inflammatory activity at enrolment was low to moderate (median C-reactive protein 1.1 mg/L, IQR 0.6–4.5). Renal function was preserved throughout (median eGFR 92.0 mL/min/1.73 m², IQR 79.6–100.6). Available baseline disease-activity indices were consistent overall with clinically relevant, predominantly moderate inflammatory disease activity at treatment initiation (**Table 1**).

**Table 1:** Baseline characteristics of the study population (n = 36).

Variable	Overall (n = 36)
Demographics and Anthropometrics	
Age, years	51 (33–60)
Female sex, n (%)	20 (55.6%)
Body mass index, kg/m²	27.2 (23.7–30.3)
Body surface area, m²	1.85 (1.71–1.97)
Index Disease and Cardiometabolic Comorbidities	
Atopic dermatitis/prurigo nodularis, n (%)	20 (55.6%)
Rheumatoid arthritis, n (%)	10 (27.8%)
Psoriatic arthritis/spondyloarthritis, n (%)	6 (16.7%)
Disease duration, years	8.5 (4.0–22.5)
Arterial hypertension, n (%)	12 (33.3%)
Type 2 diabetes mellitus, n (%)	4 (11.1%)
Dyslipidaemia, n (%)	10 (27.8%)
Metabolic syndrome, n (%)	7 (19.4%)
Current smoker, n (%)	4 (11.1%)
Smoking exposure, pack-years	0.0 (0.0–2.0)
Disease Activity Indices at Baseline	
EASI	18.5 (14.0–24.0)
DAS28-CRP	4.3 (3.7–5.1)
DAPSA	21.0 (18.0–31.5)
BASDAI	5.0 (4.3–5.4)
Laboratory Variables	
C-reactive protein, mg/L	1.1 (0.6–4.5)
HbA1c, %	5.6 (5.4–5.9)
eGFR, mL/min/1.73 m²	92.0 (79.6–100.6)
LDL cholesterol, mg/dL	114 (98–117)
HDL cholesterol, mg/dL	52 (44–61)
Echocardiographic Variables – LV Structure	
LV mass/height^2.7^, g/m^2.7^	27.9 (23.4–35.9)
Relative wall thickness	0.30 (0.30–0.40)
Epicardial fat thickness, mm	3.0 (2.0–4.5)
Left Ventricular Systolic Function	
LVEF, %	56.0 (55.0–61.0)
Global longitudinal strain (GLS), %	−18.4 (−19.8 to −16.2)
Diastolic Function and Atrial Remodelling	
E/e′ (mean)	6.9 (6.3–7.9)
Left atrial reservoir strain, %	33.5 (27.0–38.8)

Continuous variables are presented as median (interquartile range); categorical variables as number (percentage). Echocardiographic measurements performed in accordance with ASE/EACVI recommendations and analysed offline by experienced operators blinded to clinical data. eGFR, estimated glomerular filtration rate; GLS, global longitudinal strain; HDL, high-density lipoprotein; LDL, low-density lipoprotein; LV, left ventricular; LVEF, left ventricular ejection fraction. Disease-specific activity indices were available according to the underlying immune-mediated inflammatory disease subtype and routine clinical assessment. EASI, Eczema Area and Severity Index; DAS28-CRP, Disease Activity Score in 28 joints using C-reactive protein; DAPSA, Disease Activity index for Psoriatic Arthritis; BASDAI, Bath Ankylosing Spondylitis Disease Activity Index.

During follow-up, no systematic introduction or withdrawal of cardiovascular medications, antihypertensive therapy, lipid-lowering agents, or other treatments expected to directly influence myocardial deformation occurred.

### Longitudinal echocardiographic analysis

At baseline, conventional systolic function was preserved in all participants (median LVEF 56.0%, IQR 55.0–61.0). GLS values were consistent with subclinical myocardial functional impairment (median −18.4%, IQR −19.8 to −16.2), in the context of preserved LVEF. Structural indices, including LV mass indexed to height^2.7^ and relative wall thickness, were within non-pathological ranges. Median epicardial fat thickness was 3.0 mm (IQR 2.0–4.5). Diastolic function parameters and LA reservoir strain were not indicative of elevated filling pressures at study entry. Longitudinal echocardiographic data and statistical inference from rank-based models are reported in **Table 2**.

**Table 2:** Longitudinal echocardiographic variables and primary inference.

Variable	T0 median (IQR)	T1 median (IQR)	T3 median (IQR)	T6 median (IQR)	p-value*
**GLS, %**	**−18.4 (−19.8 to −16.2)**	**−18.9 (−20.5 to −17.6)**	**−19.3 (−20.6 to −18.0)**	**−19.2 (−20.2 to −17.9)**	**0.003**
**LVEF, %**	56.0 (55.0–61.0)	58.0 (55.0–60.5)	59.0 (55.0–60.0)	—	0.789
**LV mass/height** ^2.7^ **, g/m** ^2.7^	27.9 (23.4–35.9)	26.0 (23.5–35.3)	27.5 (22.7–36.6)	26.7 (22.7–34.2)	0.976
**Relative wall thickness**	0.30 (0.30–0.40)	0.30 (0.30–0.32)	0.30 (0.30–0.30)	0.30 (0.30–0.30)	0.892
**E/e′ (mean)**	6.9 (6.3–7.9)	7.5 (6.6–9.1)	7.4 (6.0–9.0)	8.0 (7.0–8.7)	0.026
**Epicardial fat, mm**	3.0 (2.0–4.5)	3.0 (2.0–5.2)	3.0 (3.0–5.2)	4.0 (3.0–6.0)	0.587
**LA reservoir strain, %**	33.5 (27.0–38.8)	34.0 (28.0–39.0)	35.5 (30.8–40.8)	34.0 (32.5–39.5)	0.613

Values expressed as median (IQR). Sample size varied across timepoints due to real-world follow-up availability. *P-values refer to the omnibus test of the overall time effect (primary analysis; rank-based longitudinal models). Bold p-values indicate statistical significance (p < 0.05). GLS, global longitudinal strain; IQR, interquartile range; LA, left atrial; LVEF, left ventricular ejection fraction.

### Primary endpoint: global longitudinal strain

**Figure 1:**
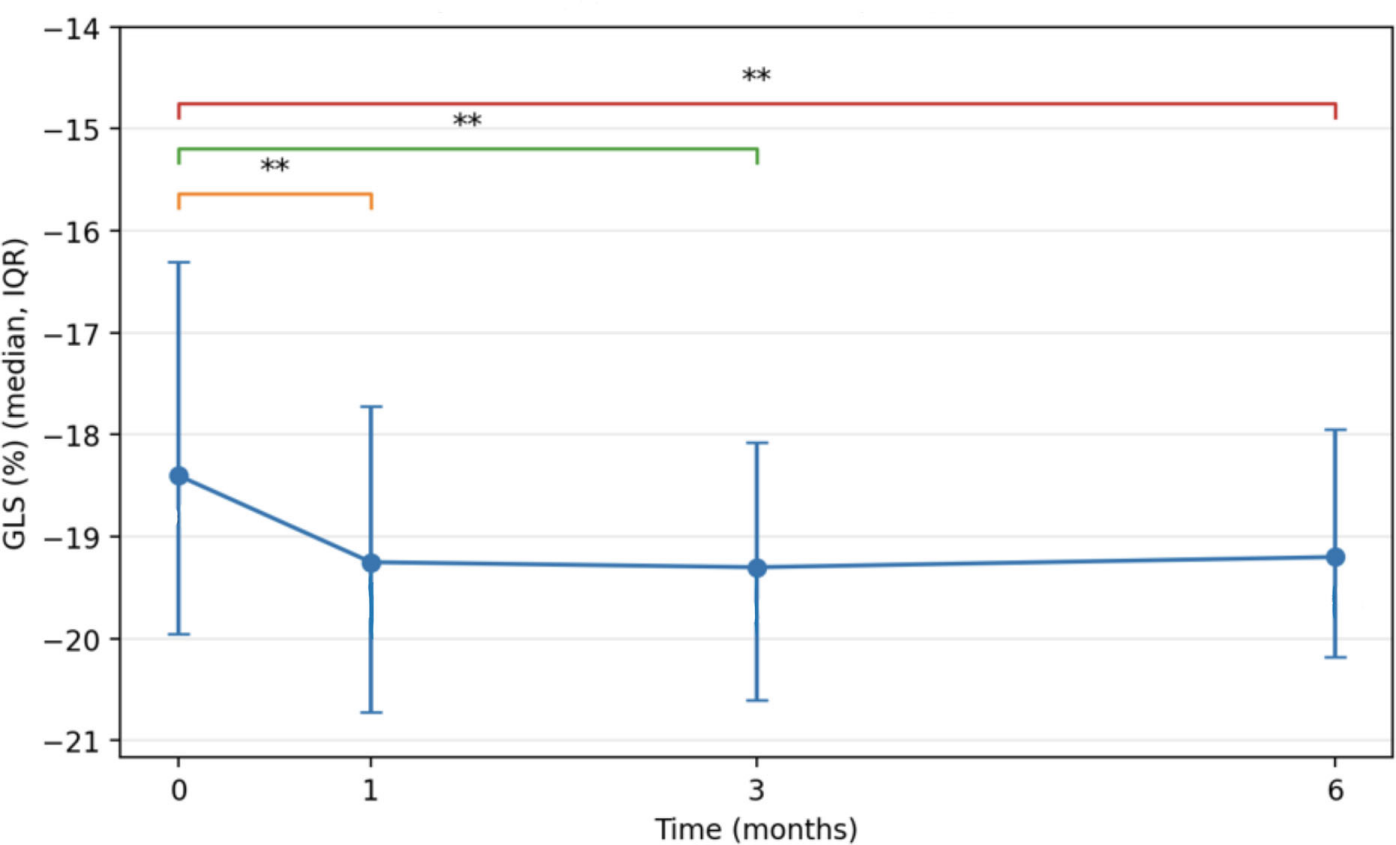
Temporal evolution of global longitudinal strain (GLS) following initiation of upadacitinib. Values are expressed as median (interquartile range). Asterisks (*) denote Holm-corrected statistically significant differences versus baseline (primary rank-based analysis): T1 p = 0.003; T3 p = 0.007; T6 p = 0.007.

GLS demonstrated a statistically significant overall time effect (omnibus p = 0.003). Pre-specified pairwise comparisons versus baseline identified a favourable myocardial deformation signal emerging early and sustained throughout follow-up: GLS improved at T1 (Holm-corrected p = 0.003), with the effect persisting at T3 (Holm-corrected p = 0.007) and T6 (Holm-corrected p = 0.007). The temporal trajectory is illustrated in **Figure 1**.

### Cardiovascular safety: conventional systolic and structural indices

LVEF showed no significant longitudinal change (omnibus p = 0.789), and no significant differences from baseline were identified at any follow-up timepoint. Structural parameters including LV mass indexed to height^2.7^ (omnibus p = 0.976), relative wall thickness (omnibus p = 0.892), and epicardial fat thickness (omnibus p = 0.587) remained stable throughout follow-up. LA reservoir strain showed no significant change (omnibus p = 0.613). The absence of significant change across these conventional indices provides no evidence of early structural or systolic harm following upadacitinib initiation.

### Secondary findings: diastolic function

**Figure 2:**
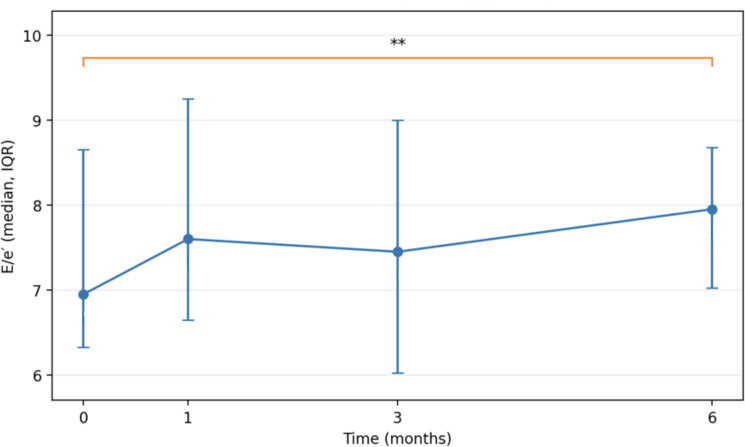
Temporal evolution of the mean E/e′ ratio following initiation of upadacitinib. Values are expressed as median (interquartile range). Asterisk (*) denotes a Holm-corrected statistically significant difference versus baseline at T6 (p = 0.008). Absolute values remained within non-pathological ranges throughout follow-up. This isolated diastolic signal was not accompanied by structural remodelling.

E/e′ exhibited a significant overall time effect (omnibus p = 0.026). Pairwise analysis demonstrated that only the increase observed at T6 reached statistical significance after Holm correction (Holm-corrected p = 0.008); differences at T1 (p = 0.113) and T3 (p = 0.346) were not statistically significant. The magnitude of the late E/e′ increase was modest, and absolute values remained within non-pathological ranges throughout follow-up. Importantly, this isolated diastolic signal was not accompanied by structural remodelling or deterioration in LA reservoir strain. The temporal evolution of E/e′ is illustrated in **Figure 2**.

### Clinical cardiovascular events

No major adverse cardiovascular events (MACE), cardiovascular hospitalization, or death occurred during follow-up. However, the study was not designed or powered for clinical cardiovascular event assessment.

## Discussion

### Principal findings

This prospective real-world study characterised the cardiovascular safety profile and early pharmacodynamic myocardial signal associated with initiation of upadacitinib, a selective JAK1 inhibitor, in 36 patients with heterogeneous IMIDs. Three principal findings merit discussion. First, no evidence of early structural or systolic myocardial harm was observed: LVEF and all LV structural indices remained stable across six months of follow-up, providing a reassuring cardiovascular profile in this selected population. Second, a favourable myocardial deformation signal as measured by GLS emerged from as early as one month following treatment initiation and was sustained through six months. Third, a modest, isolated late increase in E/e′ at six months was identified, in the absence of structural correlates, the clinical significance of which remains uncertain and warrants continued longitudinal monitoring.

### Clinical pharmacology and regulatory context

The present findings must be interpreted within the broader pharmacovigilance debate surrounding the JAK inhibitor class. Regulatory concern was substantially heightened following the ORAL Surveillance trial, which demonstrated increased risks of MACE, venous thromboembolism, and malignancy with tofacitinib compared with TNF inhibitors in patients with rheumatoid arthritis aged ≥50 years with additional cardiovascular risk factors (1). The EMA and FDA subsequently extended class-wide safety restrictions, recommending restriction or avoidance of JAK inhibitors in patients at elevated cardiovascular risk, current or recent smokers, and those with prior thromboembolic events.

Recent prospective and meta-analytic evidence has further highlighted the complexity of cardiovascular risk assessment during JAK inhibitor therapy. Popescu et al. evaluated cardiovascular risk parameters in patients with rheumatic diseases treated with JAK inhibitors, supporting the need for structured prospective cardiovascular monitoring in this therapeutic setting (32). In parallel, Partalidou et al., in a systematic review and meta-analysis comparing JAK inhibitors with TNF inhibitors in rheumatoid arthritis, emphasized that MACE and venous thromboembolism remain central safety outcomes when interpreting JAK inhibitor exposure (33). These data reinforce the rationale for pharmacovigilance-oriented studies, while also underscoring that surrogate imaging findings such as GLS cannot substitute for adequately powered clinical outcome studies.

However, the appropriate generalisation of these findings across all JAK inhibitors, all patient populations, and all disease indications has been the subject of important scientific debate. In previous work published by the corresponding author in Internal and Emergency Medicine, the cardiovascular risk associated with JAK inhibition was critically appraised (34). That analysis argued that the cardiovascular signal observed in high-risk rheumatoid arthritis populations cannot be uncritically extrapolated to lower-risk IMID patients, and that selective interpretation of safety alerts without integration of mechanistic, functional, and lipid-metabolic data risks oversimplifying a complex biological and pharmacological reality (34). In particular, inadequate management of LDL-cholesterol elevations associated with JAK inhibition may represent a key, potentially modifiable contributor to the observed cardiovascular signal — a finding with direct implications for clinical management and pharmacovigilance protocols.

The present study contributes real-world pharmacodynamic data to this ongoing debate. In an IMID cohort specifically selected to exclude high cardiovascular risk and heavy smoking — the populations most implicated in regulatory restrictions — upadacitinib initiation was not associated with any early structural or systolic myocardial harm. These findings do not challenge the validity of regulatory signals in high-risk populations but underscore the importance of refined cardiovascular risk stratification beyond class-wide restrictions, incorporating individual myocardial phenotyping alongside standard pharmacological surveillance.

### Interpretation of the global longitudinal strain signal

The favourable myocardial deformation signal observed following upadacitinib initiation — a favourable GLS trajectory from baseline to T1, sustained through T3 and T6, in the absence of LVEF change — warrants careful and strictly hypothesis-generating interpretation. GLS is sensitive to subclinical myocardial dysfunction mediated by inflammatory cytokines, which impair cardiomyocyte calcium handling, mitochondrial energy generation, and longitudinal fibre contractility without necessarily causing overt structural remodelling or LVEF decline (22–24). In the context of active IMID, chronic JAK/STAT-mediated cytokine signalling may exert a low-grade, continuous inhibitory effect on longitudinal myocardial fibre performance, detectable as GLS impairment in the presence of preserved LVEF.

**Figure 3:**
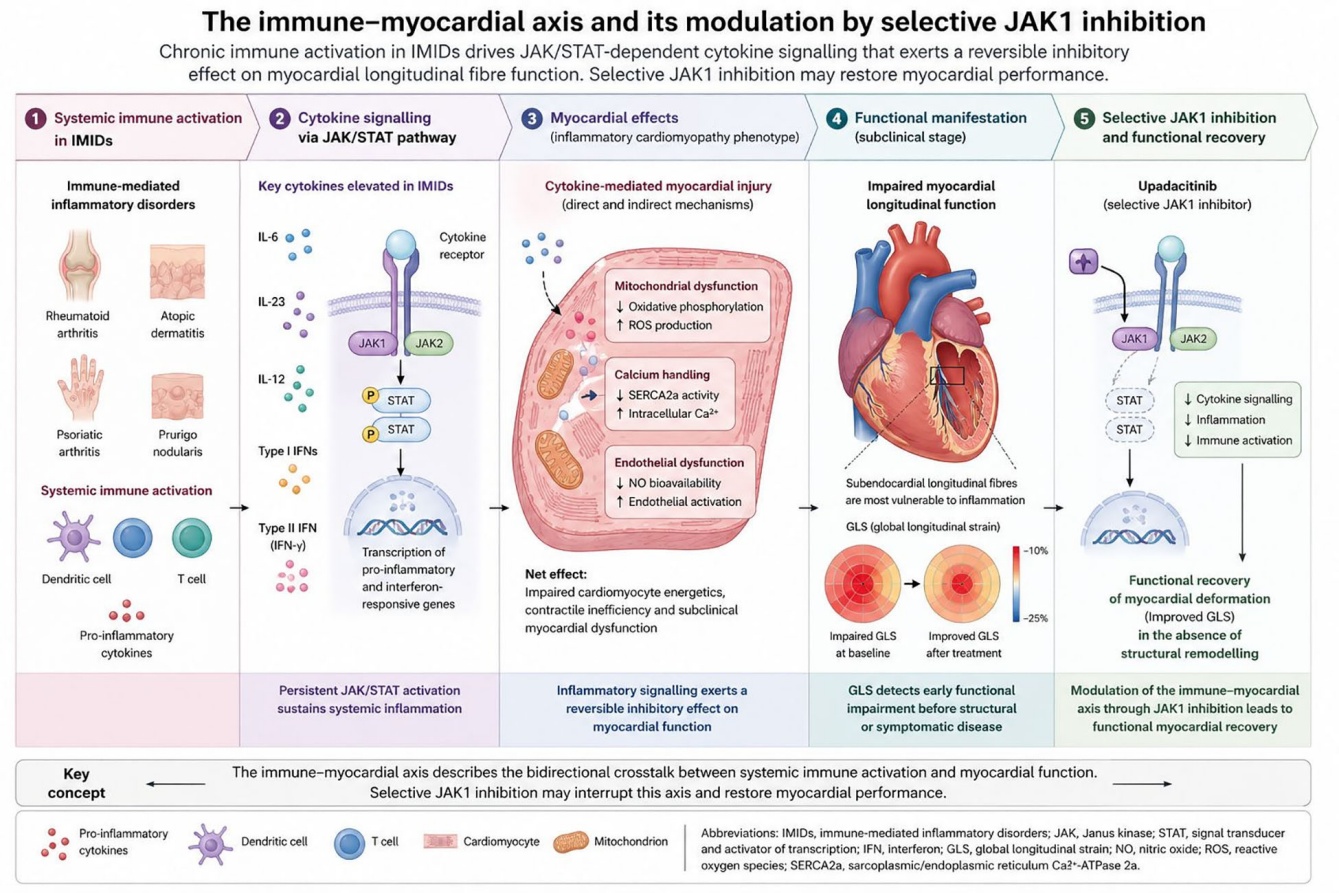
Proposed immune–myocardial axis and its modulation by selective JAK1 inhibition. Chronic immune activation in IMIDs may impair myocardial longitudinal function through JAK/STAT-mediated cytokine signalling, mitochondrial dysfunction, altered calcium handling, and endothelial activation. Selective JAK1 inhibition may attenuate inflammatory signalling and may be associated with improved myocardial deformation indices in the absence of structural remodelling.

Selective JAK1 inhibition by upadacitinib may attenuate this cytokine-mediated myocardial signalling; this is compatible with the favourable GLS trajectory observed without requiring structural geometric changes, a mechanism consistent with the observed dissociation between the GLS signal and stability of LV structural parameters. A proposed mechanistic framework of the immune–myocardial axis and its modulation by selective JAK1 inhibition is summarised in **Figure 3**. This framework supports the concept of an immune–myocardial axis in which systemic inflammatory activity modulates subclinical myocardial function (7, 9, 10).

Reduced systemic inflammation should not necessarily be interpreted as a competing explanation for the observed GLS trajectory, but rather as a biologically plausible pathway through which JAK/STAT inhibition may influence endothelial and myocardial functional signalling. The early GLS trajectory may therefore reflect attenuation of cytokine-driven endothelial and myocardial functional impairment following JAK/STAT inhibition. However, in the absence of a control group and homogeneous longitudinal disease-activity measures, a direct drug-specific myocardial effect cannot be inferred.

Natural disease fluctuation and regression to the mean cannot be excluded in an uncontrolled observational study. However, the selective GLS trajectory, in the absence of parallel improvement in LVEF, LV mass, relative wall thickness, epicardial fat thickness, or LA reservoir strain, argues against a generalized nonspecific echocardiographic drift as the sole explanation.

It is essential to emphasise that the present data are observational and uncontrolled, derived from a small heterogeneous cohort without a comparator arm. Accordingly, this mechanistic interpretation is strictly hypothesis-generating, and no causal inferences can be drawn from the current dataset.

### Comparison with published literature

To date, no prospective pharmacovigilance study has evaluated serial myocardial deformation indices before and after initiation of any JAK inhibitor, precluding direct comparison. Evidence from adjacent fields provides indirect contextual support. In cardio-oncology, GLS alterations are well-established early markers of chemotherapy- and immunotherapy-induced myocardial toxicity, reliably preceding LVEF decline by several months (27). The sensitivity of GLS to inflammation-mediated cardiomyocyte injury is further supported by studies in acute myocarditis, peripartum cardiomyopathy, and sepsis-associated cardiomyopathy.

Beyond clinical cardiovascular events, recent studies have begun to explore vascular and microvascular effects of JAK inhibition. Anyfanti et al. reported non-invasive assessments of micro- and macrovascular function after initiation of JAK inhibitors in rheumatoid arthritis (35), while subsequent prospective data from the same group examined coronary microvascular perfusion during JAK inhibitor treatment (36). These studies are complementary to the present work, as they focus primarily on vascular and microvascular phenotyping, whereas our study addresses serial myocardial deformation by speckle-tracking echocardiography after upadacitinib initiation.

In the IMID literature, subclinical myocardial dysfunction measured by GLS has been documented in rheumatoid arthritis, systemic lupus erythematosus, and inflammatory bowel disease, correlating with disease activity and systemic inflammatory burden (7, 9, 10). The present study extends this evidence base by prospectively characterising the GLS trajectory following targeted pharmacological immunomodulation, filling a critical gap in the pharmacovigilance characterisation of JAK inhibitors.

### Clinical implications

These findings carry cautious but relevant implications for clinical pharmacology practice and cardiovascular pharmacovigilance. First, the absence of early myocardial harm across conventional echocardiographic indices in this selected low-to-moderate cardiovascular risk cohort is reassuring and consistent with the safety profile observed in the upadacitinib clinical trial programme. Second, the favourable GLS signal, if confirmed in larger controlled studies, may indicate that effective inflammatory control in IMID patients is associated with a favourable myocardial functional signal, a hypothesis with implications for integrated cardiometabolic management strategies.

Third, the modest late increase in E/e′ at six months warrants continued monitoring even in the absence of structural correlates, as it may represent a haemodynamic or loading effect requiring longer-term follow-up to contextualise. Fourth, the use of serial deformation imaging as a pharmacovigilance tool in patients receiving JAK inhibitors represents a clinically feasible approach that merits prospective evaluation in larger cohorts. Pending confirmatory studies, we do not advocate changes in current prescribing or monitoring practice; however, we propose that cardiovascular safety characterisation of upadacitinib in IMID populations should incorporate functional myocardial imaging alongside conventional risk stratification.

### Strengths and limitations

Strengths. This study has several methodological strengths. The prospective design with pre-specified echocardiographic endpoints and serial assessments across four timepoints provides a structured pharmacovigilance dataset not previously available for JAK inhibitors. The real-world, single-centre setting ensures ecological validity and reflects routine clinical practice conditions. The use of two-dimensional speckle-tracking echocardiography with a standardised acquisition protocol and offline analysis provides a reproducible and sensitive measure of subclinical myocardial function. Critically, echocardiographic operators were blinded to the study hypothesis and expected direction of results at the time of examination and analysis, reducing observer bias and strengthening the internal validity of strain measurements. The application of rank-based repeated-measures statistical models is methodologically appropriate for a small real-world cohort with non-normally distributed echocardiographic data.

Limitations. Several important limitations must be acknowledged. The small sample size (n = 36) limits statistical power and precludes definitive conclusions; the present findings must therefore be considered hypothesis-generating. The single-centre design restricts generalisability to other clinical settings and patient populations. The absence of a control group — either healthy volunteers or IMID patients not receiving JAK inhibitor therapy — prevents attribution of observed GLS changes to upadacitinib specifically, as opposed to natural disease course, regression to the mean, or concomitant therapeutic changes. The heterogeneity of IMIDs enrolled represents a source of biological variability that may dilute or confound group-level effects.

Several additional limitations warrant explicit emphasis. The cohort represented a small, selected population at low-to-moderate cardiovascular risk, reflecting current EMA/FDA precautionary recommendations and prescribing patterns for JAK inhibitors; this selection strategy may have introduced selection bias and limits generalizability to higher-risk IMID populations. Because disease-activity scores were disease-specific (EASI, DAS28-CRP, DAPSA, BASDAI) and subgroup sizes were small, longitudinal disease-activity trajectories were not pooled into an adjusted model, precluding formal mediation analysis of systemic inflammation. Systematic longitudinal profiling of cardiac troponin, B-type natriuretic peptide/N-terminal pro–B-type natriuretic peptide (BNP/NT-proBNP), and inflammatory biomarkers was not available, which limits the mechanistic interpretation of the GLS signal. As a surrogate functional endpoint, GLS does not equate to clinical cardiovascular outcomes, and the study was not designed or powered to assess MACE, cardiovascular hospitalization, or mortality. Finally, echocardiographic examinations were acquired by more than one operator; although standardized acquisition protocols, blinded offline analysis, and the high reproducibility of GLS measurements (intra- and inter-observer intraclass correlation coefficients) mitigate this issue, residual operator-dependent variability cannot be entirely excluded.

## Conclusion

In this prospective real-world cohort of patients with immune-mediated inflammatory disorders initiating upadacitinib, no evidence of early structural or systolic myocardial harm was identified across conventional echocardiographic indices in a selected low-to-moderate cardiovascular-risk population. A favourable GLS trajectory was observed in the absence of LVEF change; this finding is exploratory and hypothesis-generating and may reflect attenuation of cytokine-driven inflammatory/endothelial dysfunction rather than a direct cardioprotective effect. Adequately powered, controlled prospective studies integrating myocardial imaging, vascular phenotyping, biomarkers, disease-activity trajectories, and clinical cardiovascular outcomes are required to confirm these preliminary observations.

These findings contribute pharmacodynamic data to the ongoing pharmacovigilance characterisation of JAK inhibitors and support the need for refined multimodal cardiovascular risk stratification integrating functional myocardial imaging alongside conventional risk algorithms. Adequately powered, controlled, prospective studies with multimodal cardiovascular phenotyping are required to determine their implications for long-term cardiovascular outcomes in IMID patients receiving JAK inhibitor therapy.

## Data Availability

The raw data supporting the conclusions of this article will be made available by the authors, without undue reservation.
